# Identification and mapping of a recessive allele, *dt3*, specifying semideterminate stem growth habit in soybean

**DOI:** 10.1007/s00122-023-04493-w

**Published:** 2023-11-30

**Authors:** Chancelor B. Clark, Dajian Zhang, Weidong Wang, Jianxin Ma

**Affiliations:** 1 Department of Agronomy, Purdue University, 915 W Mitch Daniels Blvd, West Lafayette, IN 47907 USA; 2 Center for Plant Biology, Purdue University, West Lafayette, IN USA; 3 Present Address: College of Agronomy, Shandong Agricultural University, Tai’an, 271018 Shandong China; 4 Present Address: College of Agronomy, China Agricultural University, Beijing, 10091 China

## Abstract

**Key message:**

A locus, *dt3*, modulating semideterminancy in soybean, was discovered by a combination of genome-wide association studies and linkage mapping with multiple distinct biparental populations.

**Abstract:**

Stem growth habit is a key architectural trait in many plants that contributes to plant productivity and environmental adaptation. In soybean, stem growth habit is classified as indeterminate, semideterminate, or determinate, of which semideterminacy is often considered as a counterpart of the “Green Revolution” trait in cereals that significantly increased grain yields. It has been demonstrated that semideterminacy in soybean is modulated by epistatic interaction between two loci, *Dt1* on chromosome 19 and *Dt2* on chromosome 18, with the latter as a negative regulator of the former. Here, we report the discovery of a third locus, *Dt3,* modulating soybean stem growth habit, which was delineated to a ~ 196-kb region on chromosome 10 by a combination of allelic and haplotypic analysis of the *Dt1* and *Dt2* loci in the USDA soybean Germplasm Collection, genome-wide association studies with three subsets of the collection, and linkage mapping with four biparental populations derived from crosses between one of two elite indeterminate cultivars and each of four semideterminate varieties possessing neither *Dt2* nor *dt1*. These four semideterminate varieties are recessive mutants (i.e., *dt3*/*dt3*) in the *Dt1*/*Dt1*;*dt2*/*dt2* background. As the semideterminacy modulated by the *Dt2* allele has unfavorable pleotropic effects such as sensitivity to drought stress, *dt3* may be an ideal alternative for use to develop semideterminate cultivars that are more resilient to such an environmental stress. This study enhances our understanding of the genetic factors underlying semideterminacy and enables more accurate marker-assisted selection for stem growth habit in soybean breeding.

**Supplementary Information:**

The online version contains supplementary material available at 10.1007/s00122-023-04493-w.

## Introduction

Soybean (*Glycine max* (L.) Merr.) is the most widely grown oilseed crop in the world, with nearly 129 million hectares (ha) planted in 2021, according to the Food and Agriculture Organization of the United Nations (FAO 2021). Soybean yields have steadily increased over the past century through selective breeding and agronomic changes (Specht et al. 2014). However, these yield increases have lagged those observed in staple cereal grains such as maize, rice, and wheat, which had major “green revolution” breakthroughs precipitated by the optimization of shoot architecture for modern agricultural systems (Liu et al. 2020). As a result, understanding the genetic bases underlying key soybean shoot architectural traits is imperative for continued productivity growth in soybean.

Soybean stem growth habit is a vital trait that lies at the intersection of shoot architecture and flowering to determine soybean yield through its effects on height, node number, stress tolerance, and adaptation to diverse environments (Ping et al. 2014). This trait is defined as the duration of vegetative growth at the shoot apical meristems (SAMs) after the induction of flowering at axillary meristems elsewhere in the plant. Determinate soybeans cease vegetative growth at the SAM almost immediately upon the plant reaching its beginning flowering (R1) stage as the SAM transitions to a reproductive inflorescence that forms a characteristic terminal cluster of flowers. Indeterminate soybeans in contrast maintain vegetative growth at the SAMs for the length of the growing season as resources allow even as reproductive growth occurs simultaneously at other meristems. Semideterminate soybeans are an intermediate class in which the vegetive SAM is maintained for a period of time after R1 before transitioning to a terminal raceme like that observed in determinate varieties (Ping et al. 2014). In general, determinate soybean varieties are grown in areas where long growing seasons allow sufficient time for distinct vegetive and reproductive stages, while indeterminate soybean varieties are preferred at higher latitudes with shorter growing seasons (Clark and Ma 2023). Semideterminate soybean varieties are also useful in short-season environments and have been adopted in some regions such as northeastern China (Liu et al. 2008). They may be able to provide the advantages of determinate varieties, such as resistance to lodging, increased pod number per node, and uniformity of pod maturation, without sacrificing the flexibility of development and increased node number that makes indeterminate varieties desirable (Ping et al. 2014). The compact architecture of semideterminate soybeans echoes the semi-dwarf phenotypes utilized to launch the “green revolution” in cereal crops and consequently could be a major target for soybean improvement.

Classical genetic analysis identified two genes, *Dt1* and *Dt2,* which control stem growth habit in soybean (Bernard 1972). *Dt1* and *dt1* govern indeterminate or determinate growth, respectively, and the indeterminate allele *Dt1* is dominant over the determinate allele *dt1. Dt2* and *dt2* modulate semideterminate and indeterminate growth, respectively, in the *Dt1* backgrounds. *dt1* is recessively epistatic to *Dt2*, so that soybeans with the genotype *dt1dt1* are determinate regardless of what allele is present at the *Dt2* locus. The functional *Dt2* allele conferring a semideterminate growth habit is completely dominant over the *dt2* allele conferring an indeterminate growth habit (Bernard 1972; Ping et al. 2014). Depending on the environment and genetic background, terminal flowering at the SAM caused by the *dt1* allele may be indistinguishable from terminal flowering caused by the *Dt2* allele, so that while these are distinct phenotypic classes, in practice semideterminacy refers to stem termination in soybeans which do not carry the genotype *dt1/dt1* (Clark and Ma 2023). *Dt1* was later identified to be *Glyma.19g194300,* which is the functional ortholog of *Arabidopsis thaliana TERMINAL FLOWERING 1* (*TFL1*) encoding a phosphatidylethanolamine-binding protein (PEBP) and maintains indeterminate growth at the shoot apex (Tian et al. 2010; Yue et al. 2021). At least four recessive loss-of-function mutations in *Dt1* result in determinate growth (Tian et al. 2010). *Dt2* is *Glyma.18g273600,* which encodes a MADS-domain transcription factor that represses *Dt1* by directly binding to its promoter (Ping et al. 2014; Liu et al. 2016). *Dt2* also acts as a transcriptional activator of genes which induce flowering including *GmSOC1*, *GmAP1*, and *GmFUL* (Zhang et al. 2019).

There is considerable quantitative variation in degree of soybean stem termination within the three broad phenotypes of determinate, semideterminate, and indeterminate. We wondered whether additional genes influence the range of phenotypic values classified as semideterminate. In this study, we employed allelic analysis of soybeans in the USDA soybean Germplasm Collection and a series of genome wide association studies (GWAS) to further dissect the genetic basis underlying semideterminacy in soybeans. We focused particularly on semideterminate soybeans which did not possess the known stem termination alleles of *dt1* or *Dt2*. We then selected four of such accessions, crossed them to indeterminate cultivars, and through linkage mapping, found that *dt3*, a recessive allele(s) at a novel locus on chromosome 10, is responsible for their semideterminacy. This study provides a starting point for a more comprehensive understanding of the genetic underpinnings of stem growth habit in soybeans and will aid soybean breeders in marker assisted selection (MAS) for this key trait.

## Materials and methods

### Plant materials

A total of 67 publicly available landraces which were classified as semideterminate according to publicly available phenotypic data from the USDA soybean germplasm collection but which don’t carry known stem termination alleles (i.e., *Dt2* or *dt1*) are listed in Table S1. Five biparental mapping populations were developed and used in this study. The first consists of 270 F_2_ lines derived from a cross between the high-yielding indeterminate cultivar HS6-3976 and the semideterminate landrace PI 548406 (Richland). The second consists of 108 F_2_ lines derived from a cross between the indeterminate cultivar Williams 82 and the semideterminate landrace PI 297520 (Iregi Universal). The third consists of 108 F_2_ lines derived from a cross between the indeterminate cultivar Williams 82 and the semideterminate landrace PI 561350B (Yong ji qun zhong da dou). The fourth consists of 153 F_2_ lines and F_3_ families derived from a cross between the indeterminate cultivar Williams 82 and the semideterminate landrace PI 597403B (Krasnodar 391–89). The fifth population consists of 50 F_2_ lines derived from a cross between the indeterminate cultivar Williams 82 and the semideterminate landrace PI 248409 (Subotica).

### Mapping population development

Four of the 67 publicly available semideterminate accessions and PI 561350B were selected for crossing once the terminal raceme was visible during R2 (full flowering stage) and crossed to Williams 82 or HS6-3976. Crossing was conducted in the field at the Purdue Agronomy Center for Research and Education (ACRE), with F_1_ seeds grown in the greenhouse. Markers polymorphic between the pairs of parents were used to confirm the hybrid status of the F_1_ seeds. F_2_ plants were phenotyped twice, at the R5 (beginning seed) and R7 (Full maturity) growth stages and classified as either indeterminate or semideterminate based on, respectively, the absence or presence of a terminal cluster of pods. F_2:3_ families were planted the following year at ACRE and phenotyped in the same manner for the Williams 82 by Krasnodar 391–89 population. The 67 publicly available accessions were phenotyped in the same manner for two consecutive years in the field at ACRE.

### Genome-wide association study

Genotypic data was drawn from the Illumina Infinium SoySNP50K BeadChip database (Song et al. 2013), and phenotypic data was drawn from the USDA Germplasm Resources Initiative (GRIN) accessed through SoyBase. 1000 accessions were randomly selected using R. Phenotypes were set as determinate = 0, semideterminate = 1, and indeterminate = 2. GWAS was performed with the TASSEL 5 software (Bradbury et al. 2007) using the Mixed Linear Model (MLM). The 170 indeterminate accessions used in GWAS after filtering out *dt1* were randomly selected using R. P-values were considered significant at a threshold of 0.05 divided by the population number, n, within each GWAS.

### DNA isolation and bulked segregant analysis

In general, 10–12 of the most typical semideterminate and indeterminate F_2_ individuals were selected from each F_2_ population. Plants were harvested in individual bags and 25 seeds for each of the selected plants and the parent lines were germinated in a Petri dish for DNA isolation. Hypocotyls were cut from 15 to 20 germinating seeds for each plant. Hypocotyls for the selected plants of each phenotype within each cross were bulked and treated as a single sample. DNA was isolated using a standard CTAB method modified from Mace et al. (2003). Bulked and parent samples were genotyped using the BARCSoySNP6K (Song et al. 2020) which contains about 6000 single nucleotide polymorphisms (SNPs). Regions were identified in which the bulked sample was homozygous for one of the two parent genotypes according to the principle of BSA.

### Linkage analysis

The population derived from Williams 82 and the semideterminate landrace Krasnodar 391–89 was used for linkage analysis. Insertion deletion (InDel) markers polymorphic between the parents were used for genotyping. Linkage analysis calculations were performed according to the Kosambi function using QTL IciMapping. The InDel primers used in this study are listed in Table S9.

## Results

### GWAS and allelic analysis identified semideterminate *Dt2*soybean varieties possessing neither *dt1* nor *Dt2*

**Fig. 1:**
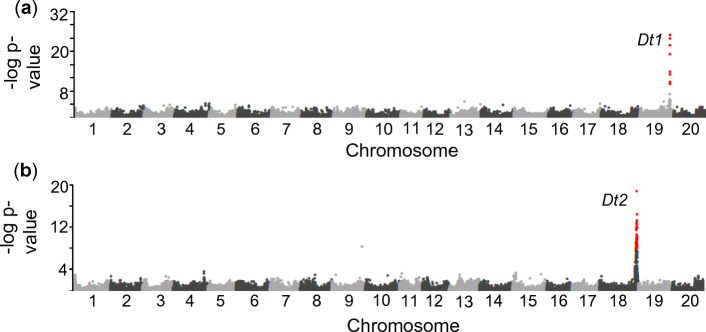
Genome wide association studies for stem growth habit. **a** analysis of 990 random accessions reveals a single major locus on chromosome 19 corresponding to *Dt1*. Significant SNPs at the Dt1/dt1 locus are colored red. **b** analysis of 170 semideterminate lines remaining after filtering out dt1 loss of function alleles paired with 170 random indeterminate lines reveals the *Dt2* locus on chromosome 18. The peak markers at this region were used to filter out semideterminate accessions carrying the functional *Dt2* allele conferring semideterminate stem growth and identify those accessions potentially carrying novel stem termination alleles. Significant SNPs at the Dt2/dt2 locus are colored red

Of the 17,376 accessions within the USDA Soybean Germplasm Collection with available stem growth habit phenotypic data, 8629 are classified as determinate, 7439 indeterminate, and 1308 semideterminate (Fig. S1). To explore whether any loci other than the previously described *Dt1* and *Dt2* are responsible for the semideterminate phenotype, we selected 990 random accessions (Table S1) from the USDA collection and conducted a genome wide association study (GWAS) for growth habit using the Illumina Infinium SoySNP50K BeadChip database (Song et al. 2013). This revealed a single major peak on chromosome 19 corresponding to the *Dt1* locus (Fig. 1a) with the strongest associated SNP marker being ss715635425 (Gm19: 45,204,441), about 19 kb downstream of the *Dt1* gene based on the Williams 82 reference genome second annotation (Fig. 1a).

We next turned to all accessions classified as semideterminate. To exclude those accessions where the semideterminate stem growth habit classification resulted from the loss-of-function alleles of *dt1*, we removed all lines which had the *dt1* alleles associated with determinate growth according to the GWAS at the SNP markers ss715635415 (Gm19:45,141,000) and ss715635425 (Gm19: 45,204,441). A total of 170 semideterminate accessions remained after this filtering process. We then randomly selected 170 accessions classified as indeterminate, paired these with the 170 remaining semideterminate accessions (Table S2), and again conducted a GWAS for stem growth habit. This second GWAS revealed a single major peak on chromosome 18 at the *Dt2* locus, with the most significant SNP being marker ss715632223 (Gm18: 55,622,046) about 16 kb upstream of the annotated transcription start site for the *Dt2* gene (Fig. 1b).

We then filtered out those semideterminate accessions which carried the dominant semideterminate *Dt2* allele in the same manner as was done for the *Dt1* locus, keeping only those which did not have the *Dt2* allele at SNP 55622046 associated with semideterminacy. A total of 74 accessions classified as semideterminate remained after filtering out of *dt1* and *Dt2* (Table S3). A third GWAS consisting of these 74 semideterminate accessions and 74 randomly selected indeterminate accessions failed to identify any significant loci. Nonetheless, we proceeded to evaluate the 74 lines. Sanger sequencing of the causal SNP sites for *dt1* and the SNPs associated with the *Dt2* haplotype reported in our earlier study (Ping et al. 2014) was performed for each of the 74 lines. This showed that four of the 74 accessions likely carried the semideterminant *Dt2* allele and three of the accessions carried the determinant *dt1* allele, leaving 67 accessions classified as semideterminate but not carrying stem termination alleles at either of the two known loci.

**Fig. 2:**
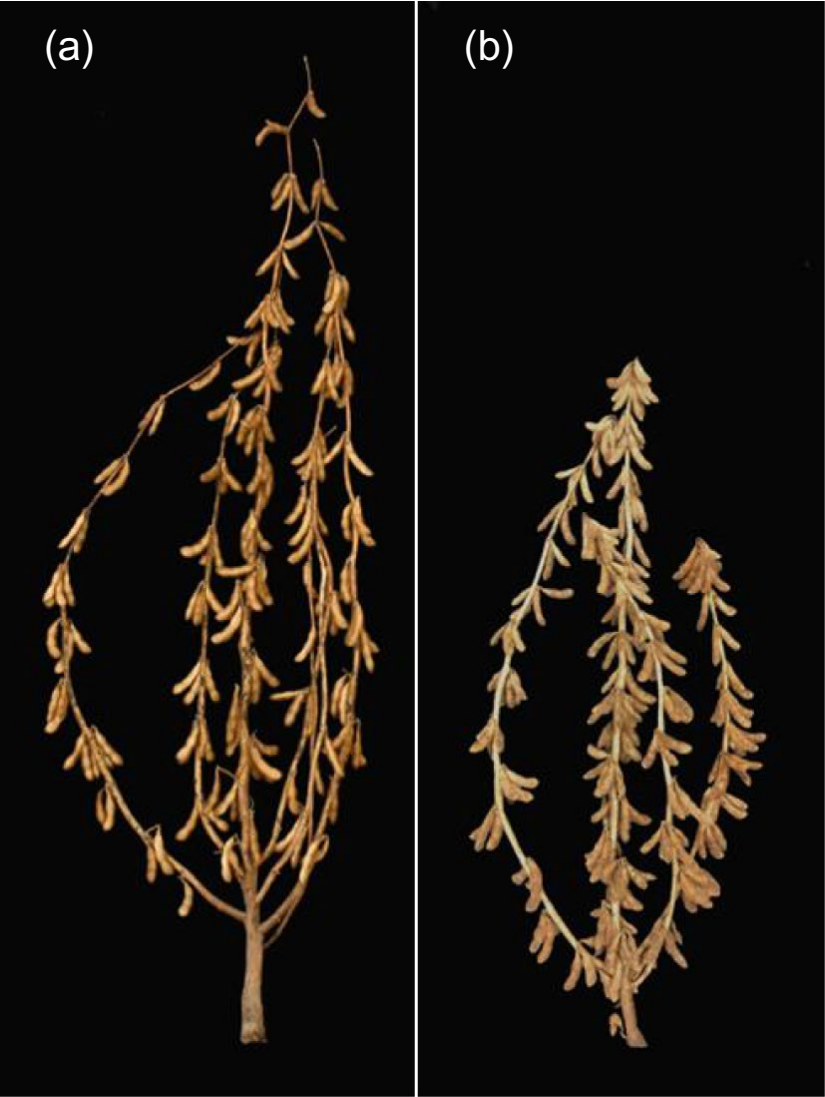
Exemplification of phenotypic difference in stem growth habit. **a** Williams 82, a typical indeterminate cultivar **b** Richland, one of one of the 37 typical semideterminate lines not carrying *dt1* or *Dt2*

When planted in the field, 37 of the 67 “semideterminate” accessions displayed typical semideterminate phenotypes (Fig. 2; Table S3). The remaining 30 accessions are not necessarily classified incorrectly, as the semideterminate phenotype of soybean landraces adapted to lower latitudes may be masked by their late flowering time in the midwestern USA where this study was conducted. The 37 lines displayed variation in degree of stem termination but were visually indistinguishable from semideterminate lines known to carry the *Dt2* allele.

### Inheritance pattern of semideterminacy from the semideterminate accessions

Three of the 37 semideterminate accessions (Krasnodar 391–89, Subotica, and Iregi Universal) were crossed to the indeterminate cultivar Williams 82 and one, Richland, was crossed to the indeterminate cultivar HS6-3976. The F_2_ progeny plants were screened in the field and classified as semideterminate or indeterminate (Table 1). In the (Williams 82 × Subotica) population, 88 individuals were classified as indeterminate and 20 as semideterminate (χ^2^ for 3:1 = 2.420, *p* = 0.1198). In the (Williams 82 × Krasnodar 391–89) population, 110 individuals were classified as indeterminate and 43 semideterminate (χ^2^ for 3:1 = 0.786, *p*-value = 0.3752). In the population derived from crossing Williams 82 with Iregi Universal, 40 individuals were classified as indeterminate and 10 were classified as semideterminate (χ^2^ for 3:1 = 0.66667, *p* = 0.4142). Finally, in the population derived from crossing HS6-3976 with Richland, 199 individuals were classified as indeterminate and 71 semideterminate (χ^2^ = 0.242, *p*-value = 0.6228). These observations suggest that the semidetermancy carried by each of these four semideterminate accessions is likely to be controlled by a single gene, and that, distinct from *Dt2*-mediated semideterminacy, the semideterminacy in these four accessions was caused by naturally occurring recessive mutations.

**Table 1:** Phenotypic segregation for stem growth habit in four F_2_ populations

F_2_ population	Phenotypes			
	Indeterminate	Semideterminate	χ^2^ (3:1)	*P* value
Williams 82 × Krasnodar 391–89	110	43	0.786	0.3752
Williams 82 × Subotica	88	20	2.42	0.1198
Williams 82 × Iregi Universal	40	10	0.667	0.4142
HS6-3976 × Richland	199	71	0.242	0.6228

### Discovery of a new locus, *Dt3*, at which recessive mutation (s) resulted in semideterminacy

**Fig. 3:**
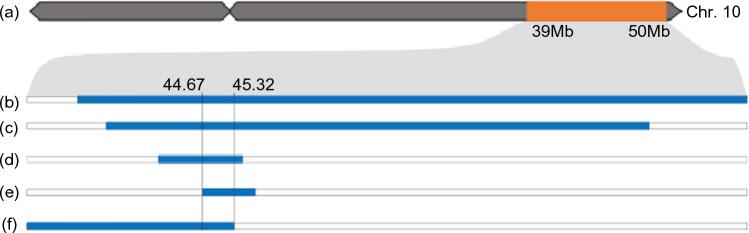
Bulked segregant analysis (BSA) of five biparental mapping populations. **a** The location of the dt3 region on the long arm of chromosome 10 highlighted by the orange bar. **b** The location of the *dt3* region in the HS6-3976 × Richland population defined by BSA. **c** The location of the dt3 region in the Williams 82 × Subotica population defined by BSA. **d** The location of the dt3 region in the Williams 82 × Iregi Universal population defined by BSA. **e** The location of the *dt3* region in the Williams 82 × Krasnodar 391–89 population defined by BSA. **f** The location of the dt3 region in the Williams 82 × PI 561350B population defined by BSA

Bulked segregant analysis (BSA) of the four biparental populations revealed overlapping regions on the long arm of chromosome 10 from the semideterminate parents are associated with semideterminacy (Fig. 3a). The regions revealed by the (HS6-3976 × Richland), (Williams 82 × Subotica), (Williams 82 × Iregi Universal), and (Williams 82 × Krasnodar 391–89) populations were bounded by SNP markers at 40.58 and 50.04 Mb (Fig. 2 b, Table S7), 41.65 and 47.7 Mb (Fig. 2 c. Table S6), 43.02 and 45.55 Mb (Fig. 2 d, Table S6), and 44.67 and 45.83 Mb (Fig. 2e, Table S6), respectively, according to the Williams 82 reference genome. Together, these observations suggest that the semideterminacy in the four parental lines is controlled by recessive mutations that likely occurred at the same locus, designated *Dt3*.

### An Inheritance pattern suggests a semideterminate accession carries both *Dt2 *and *dt3*

We also crossed PI 561350B, one of the semideterminate lines identified by our screen but which was found to carry the functional allele of *Dt2,* to Williams 82. We found that 91 F_2_ individuals derived from the two parent lines were classified as semideterminate and 17 as indeterminate. Instead of a 3:1 (semideterminate to indeterminate) ratio (χ^2^ = 4.938 *p*-value = 0.0263), we observed a 13:3 (semideterminate to indeterminate) ratio (χ^2^ = 0.642, *p*-value = 0.4230). This ratio can be explained by the hypothesis that semideterminacy in PI 561350B is determined by two loci, *Dt2* and *dt3*. As expected, BSA of the (Williams 82 × PI 561350B) F_2_ population revealed that the semideterminacy in PI 561350B is associated with 37.69–45.32 Mb (i.e., the *dt3* region) on chromosome 10 (Fig. 2f, Table S6), and with the 54.53–57.90 Mb (i.e., the *Dt2* region) on chromosome 18. These observations support our hypothesis that PI 561350B carries both the *Dt2* and *dt3* alleles for semideterminacy.

### Linkage map of the *dt3* locus

**Fig. 4:**
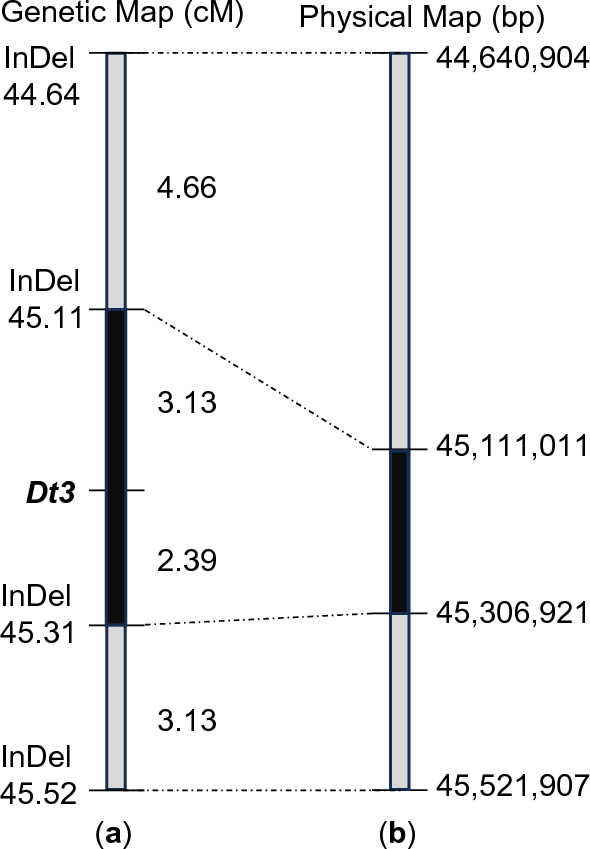
Genetic and physical maps of the Dt3 region. **a** Genetic map of the Dt3 region constructed based on linkage analysis. InDel markers are listed on the left side and the genetic distance (centimorgan, cM) between adjacent markers are shown on the right side of the map. **b** Physical positions of the molecular markers on chromosome 10 according to the soybean reference genome (Wm82.a2)

To further map the location of the *dt3* locus, the 153 individual plants of the (Williams 82 × Krasnodar 391–89) F_2_ population were genotyped (Table S8) using four InDel markers polymorphic between the two parent lines within the region defined by BSA (Fig. 3). F_2:3_ families of 10–15 plants derived from the F_2_ individuals were used to obtain more accurate phenotypes than could be obtained from individual F_2_ plants. Linkage analysis integrating the genotypic and phenotypic data placed *Dt3* in the ~ 195 kb region between two Indel markers at 45.111 and 45.306 Mb of chromosome 10 according to the Williams 82 reference genome (Fig. 4). *Dt3* is situated 3.13 centiMorgans (cM) from InDel 45.111 and 2.39 cM from InDel 45.306 (Fig. 4).

### Candidate genes for *Dt3* in the mapped region of Williams 82

There are 21 annotated genes within the defined ~ 195 kb *Dt3* region of Williams 82, some of which are known to be involved in flowering or related pathways. These include *Glyma.10G219500*, whose best matched homolog in *A. thaliana* encodes CLEAVAGE STIMULATION FACTOR 64 (CSTF64) and facilitates the silencing of the floral suppressor FLOWERING LOCUS C (FLC) (Liu et al. 2010). Another gene in this region, *Glyma.10G221200,* whose best-matched homolog in *A. thaliana* encodes a Choline/ethanolamine kinase, participates in the biosynthesis of phosphatidylethanolamine and phosphatidylcholine. Modifications to the biosynthesis of phosphatidylcholine have been implicated in flowering time changes, in part because the floral integrator FT can bind to them (Nakamura et al. 2014; Susila et al. 2021). Additionally, *Glyma.10G221500,* which sits at the edge of the mapped region, is the maturity locus *E2* and encodes a GIGANTEA-like protein (Watanabe et al. 2011) although variation in this gene has not previously been associated with changes to stem growth habit.

## Discussion

We identified a novel gene, *dt3*, specifying semideterminate growth habit in four investigated soybean varieties which do not carry *Dt2* or *dt1*. The inheritance pattern in this study suggests that *Dt3* is necessary but not sufficient to maintain indeterminate stem growth in soybean. If this is indeed the case, we can assume that the *dt1* locus is recessively epistatic to the *Dt3* locus, so that the genotypes *dt1dt1;dt2dt2;dt3dt3* and *dt1dt1;dt2dt2;Dt3Dt3* would each be determinate. However, as we are not aware of any soybean lines which possess these two genotypes, this will need to be experimentally validated through controlled crosses. The BSA result of the cross between Williams 82 and PI 561350B indicates that the semideterminate alleles of *dt3* and *Dt2* have synergistic effects, resulting in a more strongly determinate stem growth habit than either of these mutations alone. The combined effects of the *dt3* and *Dt2* alleles however still produce a terminal stem growth habit which is less extreme than the determinate growth conferred by the *dt1* loss-of-function mutations and should still be classified as semideterminate (Table 2).

**Table 2:** Soybean stem growth habit genotypes and phenotypes (Genetic basis underlying soybean stem growth habit)

Genotype	Phenotype
*Dt1/Dt1;dt2/dt2;Dt3/Dt3*	Indeterminate
*Dt1/Dt1;dt2/dt2;dt3/dt3*	Semideterminate
*Dt1/Dt1;Dt2/Dt2;Dt3/Dt3*	Semideterminate
*Dt1/Dt1;Dt2/Dt2;dt3/dt3*	Semideterminate
*dt1/dt1;dt2/dt2;Dt3/Dt3*	Determinate
*dt1/dt1;Dt2/Dt2;Dt3/Dt3*	Determinate

Semideterminate growth habits have been described in several plant species, including tomato (*Solanum lycopersicum* L.) and two leguminous crops closely related to soybean, pigeon pea (*Cajanus cajan* Millsp.) and chickpea (*Cicer arietinum* L.) (Ambika et al. 2021; Elkind et al. 1991; Kapoor and Gupta 1991). In each of these species, two genes have been reported which modulate stem growth habit. As with soybean *Dt1*, the first of these genes possesses a dominant functional allele that maintains indeterminate stem growth*,* known as *CcDt1, CaDt1,* and *SELF PRUNING* (*SP*) in pigeon pea, chickpea, and tomato, respectively (Hegde 2011; Pnueli et al. 1998; Saxena et al. 2017). However, in contrast to soybean *Dt3* and *dt3,* which modulate indeterminate and semideterminate stem growth, respectively, in *Dt1/Dt1* backgrounds, the second stem-termination genes described in tomato, chickpea, and pigeon pea specify semideterminate or determinate growth in the recessive backgrounds of their respective *Dt1* orthologs. In chickpea and pigeon pea, the semideterminate allele of the Cc*Dt2/CaDt2* locus is dominant over the determinate allele, while in tomato, the determinate allele of *Sdt* is dominant over the semideterminate allele *sdt* (Elkind et al. 1991; Harshavardhana et al. 2019; Kapoor and Gupta 1991). The inheritance pattern of semideterminacy conferred by the recessive *dt3* allele is thus distinct not only from that modulated by the soybean *Dt2* locus but also from epistatic relationships between stem growth habit genes in other plant species.

Soybean stem growth habit is revealed to be an increasingly complicated trait, with mutations in at least three genes (*dt1, Dt2,* and *dt3*) resulting in stem termination. Our initial screening to remove the *dt1* allele suggests that a substantial proportion of those accessions classified as semideterminate actually carry loss-of-function alleles of *dt1.* These phenotypic classifications may result from the *dt1-t* alleles which confer determinate growth without reducing height (Thompson et al. 1997), or from environmentally influenced phenotypic variation. Additionally, the semideterminate classification of these lines may result from quantitative genetic variation among yet-to-be-characterized genes underlying stem growth habit. When we removed *dt1*, *Dt2* could be identified by GWAS. However, neither the *Dt3*/*dt3* locus nor any other novel locus was identified by GWAS with or without the filtering out of the semideterminate *Dt2* allele, suggesting that *dt3* is a rare allele.

There has long been an interest in modifying soybean stem growth habit to increase yield, although past studies have shown mixed results based upon environment, genetic background, and management conditions (Beaver et al. 1985; Chang et al. 1982; Cober and Tanner 1995; Foley et al. 1986; Schug et al. 2022). Zhang et al. (2019) reported chromatin immunoprecipitation sequencing (ChIP-Seq) which showed that in addition to conferring semideterminate growth habits, *Dt2* also regulates genetic pathways associated with a range of traits, particularly as a negative regulator of tolerance to drought stress. The recessive *dt3* allele may therefore prove to be superior as a source of semideterminate growth habits or as a target for modification of this trait, as it may have less negative pleiotropic effects than the *Dt2* allele while conferring the same level of stem termination. As a recessive mutation, *dt3* is amenable to CRISPR-Cas9-facilitated gene editing knockouts that could easily be used to switch various soybean cultivars from indeterminate to semideterminate. Further work is needed to pinpoint the candidate gene(s) for the *dt3* locus, understand its molecular role in modulating semideterminacy, and evaluate its effect on soybean yields toward the goal of producing optimized soybean shoot architecture.

### Supplementary Information

Below is the link to the electronic supplementary material.

Supplementary file1 (XLSX 9216 KB)

## Data Availability

All data presented in this manuscript are included in the supplemental tables. All materials are available to the public upon request and under material transfer agreement.
